# Restoring our ubiquitination machinery to overcome resistance in cancer therapy

**DOI:** 10.18632/oncoscience.600

**Published:** 2024-05-06

**Authors:** Xiaoling Li, Ping Mu

**Keywords:** androgen receptor, prostate cancer, antiandrogen resistance, UBE2J1, ubiquitination, protein degradation, PROTAC

The ubiquitin-proteasome system (UPS) [1], which is usually responsible for regulating protein degradation, is important for cellular homeostasis, and playing a crucial role in cancer progression. Its ability to regulate the stability of proteins that drive cancer growth and survival indicates its potential as a therapeutic target. Among the UPS components, ubiquitin-conjugating enzymes, such as ubiquitin-conjugating enzyme E2 J1 (UBE2J1) [2], have emerged as key players in cancer dynamics, especially in prostate cancer (PCa) where therapy resistance is a significant challenge.

In our recent study, through a comprehensive *in vivo* library screening [2], we have identified the role of UBE2J1 in PCa, particularly its involvement in the degradation of the androgen receptor (AR) [3]. AR is a key oncogenic driver of PCa growth, making AR-targeted therapies like enzalutamide and abiraterone fundamental to current treatment strategies. However, resistance to these agents often develops quickly, leading to treatment failures and progression of the disease. This resistance is typically marked by the continued activity of AR signaling, despite the use of antiandrogens intended to block this pathway. Our research indicates that UBE2J1 is a critical E2 ubiquitin-conjugating enzyme responsible for the ubiquitination and subsequent degradation of AR. The loss of UBE2J1, which was found in 5–15% of PCa patients, dysregulates and impairs AR ubiquitination and degradation. This leads to AR accumulation, enhances AR signaling and contributes to resistance to antiandrogens. By knocking down UBE2J1 in PCa cell lines, we observed increased AR levels and enhanced therapy resistance. And the application of a novel ubiquitination-based AR degrader (Accutar Biotech, AC0176) effectively restored AR degradation in UBE2J1-deficient models, offering a new therapeutic strategy to counteract therapy resistance in PCa.

Resistance to antiandrogen therapy in PCa remains a significant challenge in the management of this deadly disease [4]. The known mechanisms responsible for this resistance includes increased tumor heterogeneity [5], lineage plasticity [6, 7], mutagenesis and genomic alterations [2], epigenetic rewiring [8], and the complex interactions within the tumor microenvironment [9, 10]. Despite these variables, approximately 50% of patients resistant to treatment exhibit restored AR signaling. This underscores the potential therapeutic value of specifically restoring or enhancing AR degradation. Our study highlights the crucial role of UBE2J1 in regulating AR degradation and suggests that restoration of AR degradation as a potential therapeutic approach to overcome resistance in PCa.

The role of UBE2J1 extends beyond PCa. For instance, in colorectal cancer, UBE2J1 suppresses tumor progression by promoting the ubiquitination and degradation of RPS3, thereby inhibiting the NF-κB signaling pathway [11]. This pathway is known for its role in inflammation and cancer progression, suggesting that the tumor-suppressive role of UBE2J1 could be leveraged in various cancer types. Therefore, the implications of targeting UBE2J1 in cancer therapy are substantial. Understanding the specific roles of ubiquitin-conjugating enzymes in cancer allows researchers to develop more targeted therapies that address the unique molecular characteristics of different tumors.

Looking forward, the utilization of ubiquitination-based AR degraders, combined with existing therapeutic strategies such as antiandrogens, holds promise for enhancing treatment efficacy and overcoming drug resistance by restoring AR degradation. Additionally, investigating the role of UBE2J1 in other AR-dependent cancers, such as breast cancer, could broaden the potential application of AR degraders in these malignancies. Given that various mechanisms, many of which are AR-independent, drive resistance in patients with advanced PCa, it is essential to identify those who have developed antiandrogen resistance due to impaired AR degradation. Therefore, employing UBE2J1 as an early biomarker for therapy response presents an opportunity to refine patient selection for specific treatments, ensuring that targeted therapies are administered to those most likely to benefit.

The current landscape of PROTAC (proteolysis-targeting chimeras)-type protein degrader in treating various cancers, particularly for PCa, marks a significant advancement in offering novel strategies to overcome resistance to conventional treatments. These bifunctional molecules leverage the natural protein degradation pathways to selectively degrade key oncogenic proteins, such as AR and Estrogen Receptor (ER). Studies on compounds like ARD-61 and ARD-266 have demonstrated promising results in both enzalutamide-sensitive and -resistant PCa models, showing a remarkable ability to reduce AR-regulated gene expression and inhibit tumor growth [12, 13]. PROTAC-type degraders have also shown efficacy in other cancers, such as breast cancer and T-cell acute lymphoblastic leukemia (T-ALL), where they disrupt critical pathways and reduce tumor progression effectively. For example, ARV-825 targets BET (Bromodomain and Extra-Terminal) proteins in T-ALL, leading to significant anti-tumor activity [14]. This broad applicability and effectiveness underscore the transformative potential of PROTAC-type protein degraders in targeting previously “undruggable” proteins and expanding new avenues for clinical intervention across diverse cancer types, thus revolutionizing oncological treatment by addressing drug resistance and enhancing therapeutic outcomes.

In conclusion, the identification of ubiquitin-conjugating enzymes like UBE2J1 and the innovative deployment of PROTAC-type AR degraders are crucial in combating PCa and overcoming therapeutic resistance. These methods destabilize proteins crucial for cancer progression, offering a way to tackle disease mechanisms at a molecular level. As the scientific community advances these complex technologies, the potential for transformative cancer treatments is vast. Continued dedication to this research and its application in clinical settings is key to unlocking the potential of these innovative strategies, ushering in a new era of cancer therapy.
